# Microcirculatory alterations are more severe in anemic than in ischemic hypoxia

**DOI:** 10.1186/2197-425X-3-S1-A413

**Published:** 2015-10-01

**Authors:** G Ferrara, VS Kanoore Edul, E Martins, HS Canales, C Canullán, G Murias, MO Pozo, C Ince, A Dubin

**Affiliations:** Facultad de Ciencias Médicas, Universidad Nacional de La Plata, Cátedra de Farmacología Aplicada, La Plata, Argentina; Academic Medical Center, University of Amsterdam, Translational Physiology, Amsterdam, the Netherlands

## Introduction

The intestinal mucosal-arterial PCO_2_ (ΔPCO_2_) remains remarkably stable in anemic hypoxia suggesting that the villi perfusion is well-maintained^1^. The microcirculation, however, has been insufficiently studied in extreme hemodilution.

## Objectives

To assess intestinal microcirculation during progressive hemorrhage and hemodilution.

## Methods

Sheep were assigned to either stepwise bleeding (n = 8) or blood exchange with HES 130/0.4 (n = 8). A sham group (n = 8) was also studied. Oxygen transport and consumption were measured by expired gases analysis, microcirculation with SDF-technology, and ΔPCO_2_ by air capnometry.

## Results

In the last step, there were similar reductions in systemic and intestinal oxygen transports and consumptions, and increases in respiratory quotient and lactate, in ischemic and anemic hypoxia compared to sham group. ΔPCO_2_ only increased in ischemic hypoxia (25 ± 10, 5 ± 6, and 5 ± 6 mm Hg, *P* < 0.01). Superior mesenteric artery blood flow decreased in ischemic hypoxia and increased in anemic hypoxia (138 ± 55, 524 ± 99, and 325 ± 112 mL/min, *P* < 0.0001), but mucosal and serosal microcirculations were more severely altered in anemic than in ischemic hypoxia.

## Conclusions

Although intestinal serosal and mucosal microcirculations were severely compromised in anemic hypoxia, the ΔPCO_2_ did not increase. The lack of change in ΔPCO_2_ cannot be ascribed to the preservation of villi perfusion. These findings might be explained by blood flow redistribution toward submucosal and muscular layers.

## Grant Acknowledgment

Supported by the grant PICT-2010-00495, Agencia Nacional de Promoción Científica y Tecnológica, Argentina.

**Figure 2:**
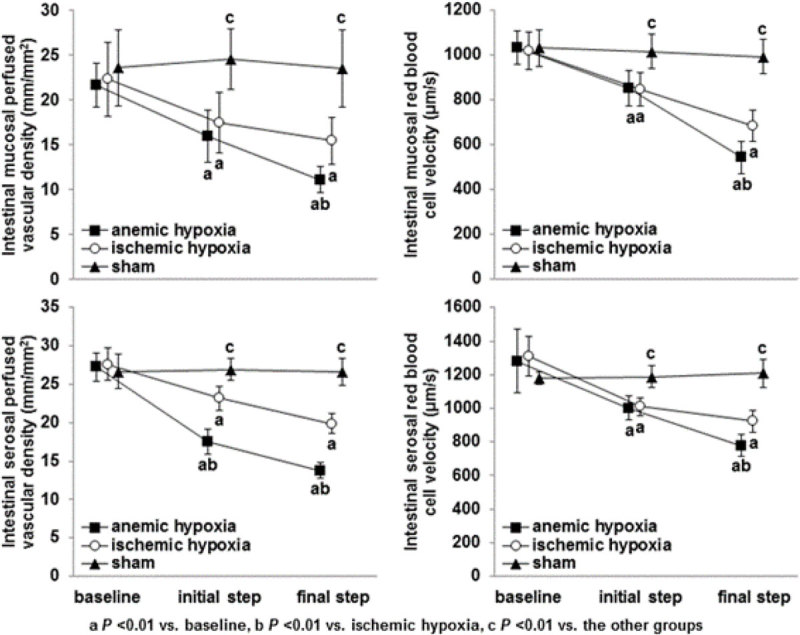
**Intestinal microcirculation.**
